# Occupational Exposures in an Equestrian Centre to Respirable Dust and Respirable Crystalline Silica

**DOI:** 10.3390/ijerph16173226

**Published:** 2019-09-03

**Authors:** Kathleen Bulfin, Hilary Cowie, Karen S. Galea, Alison Connolly, Marie Ann Coggins

**Affiliations:** 1 Centre for Climate and Air Pollution Studies, School of Physics and Ryan Institute, National University of Ireland Galway, University Road, H91 CF50 Galway, Ireland (K.B.) (A.C.); 2 Centre for Human Exposure Science, Institute of Occupational Medicine, Edinburgh EH14 4AP, UK (H.C.) (K.S.G.)

**Keywords:** respirable crystalline silica, respirable dust, occupational lung cancer, occupational exposure

## Abstract

Sand-based products are regularly used as footing material on indoor equestrian arenas, creating a potential occupational exposure risk for respirable crystalline silica (RCS) for equestrian workers training and exercising horses in these environments. The objective of this study was to evaluate an equestrian worker’s personal RCS and respirable dust (RD) exposure. Sixteen personal full-shift RD measurements were collected from an equestrian worker and analysed for RD, quartz and cristobalite. Geometric mean exposures of 0.12 mg m^−3^ and 0.02 mg m^−3^ were calculated for RD and RCS concentrations, respectively. RCS exposures of between 0.01 to 0.09 mg m^−3^ were measured on days when the indoor arena surface was not watered, compared to lower exposures (<LOD-0.03 mg m^−3^) on days when the indoor arena was watered (*p* < 0.01); however, manual watering is time intensive and less likely to be implemented in practice. This small-scale study provides new data on RCS and RD exposures among equestrian workers. RCS exposures are within the range considered to be associated with increased risk for lung cancer. The use of dust control solutions such as water suppression should be promoted for equestrian work in horse riding arenas. Equestrian workers need to receive occupational health training on the health risks associated with RCS exposure.

## 1. Introduction

The use of sand, animal feed and bedding materials can create dusty work environments for equestrian workers that tend to have a higher risk for respiratory conditions such as organic dust toxic syndrome, and bronchitis symptoms, particularly if their work is indoors [1,2,3]. Potential exposure risks from airborne pollutants including inhalable and respirable organic dusts, microorganisms, endotoxins and β-Glucans have been evaluated among equestrian workers [4,5,6]; however, less is known about exposures to inorganic dusts, such as respirable crystalline silica (RCS). RCS is a natural component of sand and associated with a range of respiratory diseases, in particular, silicosis and lung cancer [7]. Sand is regularly used in the equestrian sector as a surface or footing material in indoor and outdoor arenas, on sand gallops for training race horses, on longeing arenas and on horse walkers [8]. 

To the authors’ knowledge, there has been just one published study, which reported on horse trainers’ RCS exposures, as part of a lung cancer case report [9]. In this study, it was reported that the trainer had worked for 23 years in the sector, training 7–12 horses per day on longeing arenas covered with recycled sands. Although limited by the collection of just three exposure measurements (one area and two personal samples), 8 h time weighted average (TWA) exposure estimates for the personal sample exceeded the threshold limit value (TLV) of 0.025 mg m^−3^ for RCS set by the ACGIH [10]. This previous study highlighted a potential increased exposure risk for RCS, which could lead to the development of occupational lung cancer within this worker group. The study also suggests that there is a lack of awareness among equestrian workers of the risks of RCS exposures and limited use of exposure controls in this sector [9]. 

Choice, maintenance and age of the footing material play a significant role in the generation of particulate concentrations in indoor arenas. Maintaining a clean (manure free) well mixed moist footing material are some of the control measures recommended to reduce airborne particulates in riding arenas [11,12,13]. A recent study compared the release of airborne particulate concentrations (PM10) with footing moisture content and density for three different footing materials, sand, sand fibre mix, and sand wood chips. Particulate concentrations (PM10) from sand-fibre-based footing materials were over five times greater than concentrations for either sand or the sand wood mix. The density and moisture content of the sand fibre footing were identified as important factors influencing particulate generation. Regular watering and grooming of the footing materials to prevent separation and shifting of the materials was recommended to reduce the release of airborne particles [11].

Further exposure data is needed to characterise exposures to RCS within this occupational group, to highlight exposure risks and to promote the use of exposure controls within the sector. The objective of this study was to characterise respirable dust (RD) and RCS exposures among equestrian workers working in an Irish equestrian centre. 

## 2. Materials and Methods

One small to medium sized Irish equestrian centre, managed and operated by a self-employed worker was recruited to participate in the study, over the summer period of 2018. The centre stabled, on average, 30 horses for training and a further 15 horses for riding lessons, had one indoor arena (approximate area of 6000 m^2^) and two outdoor arenas (each had an approximate area of 10,000 m^2^), all surfaced using silica sand and shredded carpet mix (which is sand mixed with polypropylene, polyester and polyurethane fibres shredded into pieces <30 mm in length). The indoor arena was housed under the same roof as the tacking and grooming area, it had entrance sliding doors on two side walls, which were closed during the surveys. There was no mechanical ventilation. Horses were brought into the arena via the tacking and grooming area. The building also contained a small fully enclosed room under the same roof, which functioned as a canteen. The room had windows, which opened onto the arena area; however, they were rarely opened. To reduce dust levels in the indoor arena, occasionally, the surface was dampened using a water hose mounted on a ladder (approximately 3 m high), which was moved around the arena for a maximum of one hour. However, this water suppression regime was rarely performed and only if there were no competing work tasks to be performed at the centre. A convenience sampling approach was followed when collecting personal exposure data and measurements were collected when the worker performed their normal daily duties. The worker was sampled at standing height (1.5 m), apart from when they were grading/raking when then were at sitting height. Typical work duties included;

- cleaning horse stables,
- longeing horses (which involves the horse, attached to a lunge line, moving around the trainer),
- loose/free jumping 2–4 young horses per day (involves jumping a horse without a rider to practice the horses jumping skills),
- delivering riding lessons both indoors and outdoors,
- grooming, tacking and untacking horses which was always performed in the indoor arena,
- grading/raking the surface of both indoor and outdoor arenas. Surface grading is required to maintain a good workable footing material, which over time, becomes compacted due to horse traffic. In this study, the surface was graded using a rake attached to an open top tractor which was driven around the arena.

Working with the horses when longeing, free jumping or during riding lessons and raking the arena surface lead to increased dispersion of the footing material and visible clouds of dust in the arena. Personal breathing zone samples were collected using a personal sampling pump (Sidekick; SKC Ltd., Dorset, UK) with Higgins-Dewell cyclone (Casella, Bedford, UK) and 25 mm, 5 µm pore size PVC filters. Pumps were pre-calibrated at a flow rate of 2.2 L per minute (L min^−1^) using a primary airflow meter (DryCal^®^ DC Lite; BIOS International, Butler, NJ, USA). The researcher collected contextual information to support all samples collected, including time spent on potentially high-risk exposure work tasks (tasks which generated visible clouds of dust in the work area). RD samples were collected and analysed gravimetrically according to HSE MDHS 14/4 [14], the limit of detection (LOD) for RD was 0.05 mg. Both quartz and cristobalite was quantified on each sample using X ray diffraction using a Bruker D2 Phaser X ray Diffractometer with Bruker Diffrac. DQuant 1 software following HSE MDHS 101/2 [15]. Sample analysis was performed at the Institute of Occupational Medicine, Edinburgh who are accredited for XRD analysis by the United Kingdom Accreditation Service (UKAS).

Summary statistics were calculated using SPSS version 26 [16], a concentration data was log normally distributed, a paired *t*-test was used to compare RD and RCS exposures on days when the indoor arena was watered to when it was not watered. The results were compared with the Irish Occupational Exposure limit value for RCS, 0.1 mg m^−3^ [17] and the recommended comparison guideline for low-toxicity respirable dust, 1.0 mg m^−3^ [18]. Where sampling times exceeded 8 h, exposure concentrations were adjusted to an 8 h reference period [19]. Estimates of relative risk for lung cancer were calculated using RCS exposure data and log linear response curves derived by Steenland et al. [20]. 

## 3. Results

A total of 16 personal exposure measurements were collected from the one equestrian worker over the period of June–August 2018. The worker was sampled for the full work shift, including short break periods which were always spent in the arena canteen, lunch breaks (30 min) were spent off-site and not included in the measurement. Sampling times ranged from 480–540 min and the worker spent between 75% and 85% of their time working in the indoor arena or nearby during the measurement period.

### Exposure Results

Individual personal RD and RCS concentrations (mg m^−3^) are presented in Table 1. Table 1 also provides a summary of the work activities undertaken during each of the measurement periods, whether water suppression was applied in the indoor arena and also, outdoor weather conditions. The outdoor weather conditions were dry on 14 of the 16 days surveyed; there were light rain showers on days 8 and 9. On four of the measurement days, the surface of the indoor arena was sprayed with water in the morning, and on another four days, it was sprayed in the morning and afternoon. 

Cristobalite was not detected in any sample and so the RCS results reflect quartz exposure. Two of the sixteen personal samples had non-detectable levels of RD and RCS (samples 15 and 16). Sample geometric mean (GM) and geometric standard deviations (GSD) were calculated by substituting < LOD values with half the analytical LOD, this being 0.025 mg for RD and 0.005 mg for RCS [21]. GM (GSDs) of 0.124 mg m^−3^ (2.15) and 0.025 mg m^−3^ (2.43) were calculated for RD and RCS concentrations, respectively (range; <0.05 to 0.30 mg m^−3^ (RD) and <0.01 to 0.08 mg m^−3^ (RCS). 

There was a strong positive correlation between RD and RCS concentrations (*p* < 0.05). There was a significant difference (*p* < 0.01) between concentrations of both RCS and RD exposures on days when the indoor arena was watered and on days when no watering was performed.

## 4. Discussion

Personal exposure data for both RD and RCS collected from an Irish equestrian worker are presented. To the authors’ knowledge, this is only the second study of RCS exposures among equestrian workers and although based on only 16 samples, provides a larger dataset of the personal exposures experienced by these workers. The worker wore the sampling train for the full work shift, including short break periods, which were always spent in the arena canteen. This data is required to promote awareness of the health risks from exposure to RCS within this occupational group. RD concentrations over the sampling period and when adjusted to an 8 h reference period (8 h TWA) (GM (GSD); 0.12 mg m^−3^ (2.15)) are comparable to values previously reported for horse barns [4] and below 1.0 mg m^−3^, a recommended comparison guideline for RD [18]. 

RCS exposure concentrations are less than the Irish OELV of 0.1 mg m^−3^ and the EU Carcinogens and Mutagens directive (Directive (EU) 2017/2398) proposed binding occupational exposure limit (OEL) for RCS of 0.1 mg m^−3^ (8 h TWA). RCS exposures are, however, higher than the US NIOSH recommended exposure limit (REL) of 0.05 mg m^−3^. RCS exposure concentrations (0.01 to 0.09 mg m^−3^) are comparable to those reported by Yoon et al., for a horse trainer (8 h TWAs; 0.02–0.086 mg m^−3^) who was longeing and training up to 12 horses per day in an indoor arena. In the current study, the worker regularly worked greater than an 8 h shift (9 of the 16 days), a common practice among self-employed equestrian workers. The highest 8 h TWA RCS exposure (0.09 mg m^−3^) was measured on day 10. On that day, the worker spent over 80% of their time working indoors, one of the work tasks performed on day 10 involved grading the surface of the indoor arena, which created an excessive amount of dust. 

In this study, RCS concentrations were significantly higher (*p* < 0.01) on days when watering was not performed on the surface of the indoor arena, although concentrations were less than 0.1 mg m^−3^ ((GM (GSD) *n* = 8; 0.041 mg m^−3^ (2.08)). Previous research among US industrial sand workers suggest that exposures as low as 0.05 mg m^−3^ can present an increased lung cancer risk, which is further increased among smokers [20]. Yoon et al. [9] estimates a lifetime excess risk for lung cancer for an equestrian worker at age 74 of 0.077%–0.090% due to exposure to 0.02–0.086 mg m^−3^ RCS with a 15-year lag time. The relative risk for lung cancer mortality associated with 40 years exposure (with a 15-year lag period), at the concentrations measured in this current study (0.01–0.09 mg m^−3^), is estimated at between 1.004 and 1.038 (0.4% and 3.8% increase).

Given that occupational cancer is the leading cause of work-related deaths in the EU [22], further research is required to characterise RCS exposures and other potential airborne hazards created as a result of the use of different footing materials and additives including, for example, recycled textiles and synthetic carpets [23] in equestrian arenas. As exposure to mixed organic dusts, capable of inducing inflammatory reactions in the respiratory system, are common in many agricultural settings, including horse stables [6,24], future research should also include endotoxin and β (1→3)-glucan measurements. Further work should also consider particle preparations in vitro using inflammatory cells to determine the activity of the RCS and the impact of organic constituents of the aerosol. Previous work on coal mine dust suggests that other components of the aerosol may be capable of masking the reactivity of the quartz surface [25]. Studies on equine health have shown that maintaining optimal moisture content of the footing material can help manage indoor particulate concentrations in riding arenas [11]. Keeping the surface of the footing material moist and or using alternative footing materials such as sand with wood chips or wax-coated sand has the potential to reduce worker exposures to RCS but requires further evaluation. 

## 5. Conclusions

The present study, although limited by sample numbers, provides a significant way forward in characterising equestrian workers exposure to RD and RCS. Measured RCS concentrations approached the recommended Irish OEL of 0.1 mg m^−3^ for RCS, which could suggest an increased lung cancer risk for this occupational group, but since the study represents just one worker from this industry, further exposure studies are required. 

Exposure interventions, such as watering the indoor arena, reduced air concentrations of RD and RCS. However, automated watering systems would be recommended, as competing work tasks limit the time that the equestrian worker can spend on manual watering regimes. Further studies are required to promote awareness within the sector of the exposure risks associated with footing materials used on indoor equestrian arenas and the impact of increased knowledge and understanding of the risks involved. 

## Figures and Tables

**Table 1:** Summary of personal respirable dust (RD) and respirable crystalline silica (RCS) concentration data (mg m^−3^), adjusted 8 h TWA and time (mins) spent on potentially high-risk exposure tasks.

Day	Duration (min)	RD (8 h TWA)	RCS (8 h TWA)	Time Spent on Potentially High-Risk Tasks During Measurement Period							
				Water Suppression	OD Weather	Lunging/Loose Jumping	Riding Lessons	Grading ID/OD ^+^	Riding/Driving	Sweeping ID/OD ^+^	Tacking/Grooming
1	540	0.30 (0.34)	0.06 (0.07)	No	Dry	60	60	45	180		60
2	480	0.10 (0.10)	0.03 (0.03)	No	Dry	45	60		60		90
3	480	0.20 (0.20)	0.01 (0.01)	No	Dry	45			120		90
4	510	0.10 (0.11)	0.03 (0.03)	No	Dry	51	120		75		45
5	495	0.10 (0.11)	0.03 (0.03)	No	Dry	45			120	15	60
6	525	0.10 (0.11)	0.03 (0.03)	**	Dry	60	165				45
7	480	0.10 (0.10)	0.03 (0.03)	**	Dry	45			120		90
8	480	0.10 (0.10)	0.03 (0.03)	**	Wet	30	50		135	30	60
9	510	0.10 (0.11)	0.01 (0.01)	**	Wet	45	60		90		60
10	540	0.30 (0.34)	0.08 (0.09)	No	Dry	45	90	30	75	60	45
11	525	0.30 (0.33)	0.06 (0.07)	No	Dry	120			90		75
12	480	0.30 (0.30)	0.07 (0.07)	No	Dry	45	140		90		75
13	495	0.10 (0.1)	0.03 (0.03)	***	Dry	45	75		105	15	45
14	510	0.10 (0.11)	0.02 (0.02)	***	Dry	105			135		45
15	480	<0.05 *	<0.01 *	***	Dry	30	135				45
16	480	<0.05 *	<0.01 *	***	Dry	45	75		120	30	30
**GM (GSD) *n* = 16**		0.12 (2.15)	0.02 (2.43)								

OD = outdoor, ^+^ both indoors/outdoors; * sample results < limit of detection (LOD); ** morning only; *** morning and afternoon; GM = geometric mean; GSD = geometric standard deviation; concentrations < LOD substituted with ½ LOD.
